# Why do men face a higher risk of most types of cancer than women?

**Published:** 2022-09

1.

**AUGUST 3, 2022** - Understanding the reasons for sex differences in cancer risk could provide important information to improve prevention and treatment. To investigate, Sarah S. Jackson, PhD, of the National Cancer Institute, part of the National Institutes of Health, and her colleagues, assessed differences in cancer risk for each of 21 cancer sites among 171,274 male and 122,826 female adults aged 50–71 years who were participating in the NIH-AARP Diet and Health study from 1995–2011.

During that time, 17,951 new cancers arose in men and 8,742 in women. Incidence was lower in men than women only for thyroid and gallbladder cancers, and risks were 1.3- to 10.8-times higher in men than women at other anatomic sites. The greatest increased risks in men were seen for esophageal cancer (a 10.8-times higher risk), larynx (a 3.5-times higher risk), gastric cardia (a 3.5-times higher risk), and bladder cancer (a 3.3-times higher risk).

Men had an increased risk of most cancers even after adjusting for a wide range of risk behaviors and carcinogenic exposures. Indeed, differences in risk behaviors and carcinogenic exposures between the sexes only accounted for a modest proportion of the male predominance of most cancers (ranging from 11% for esophageal cancer to 50% for lung cancer).

The findings suggest that biological differences between sexes—such as physiological, immunological, genetic, and other differences—play a major role in the cancer susceptibility of men versus women.

“Our results show that there are differences in cancer incidence that are not explained by environmental exposures alone. This suggests that there are intrinsic biological differences between men and women that affect susceptibility to cancer,” said Dr. Jackson.

An accompanying editorial discusses the study’s findings and notes that a multifaceted approach needs to be in place to address sex disparities in cancer. “Strategically including sex as a biological variable should be enforced along the whole cancer continuum from risk prediction and cancer primary prevention, cancer screening and secondary prevention, to cancer treatment and patient management,” the authors wrote. “Examining and addressing sex disparities in cancer and other diseases is an ongoing quest. Bench to bedside translational studies which effectively transform the existing research findings into clinical practice is a scalable means within easy reach to achieve precision medicine and will mitigate—and may ultimately eradicate—sex disparities in cancer.”

***Link to Study: http://doi.wiley.com/10.1002/cncr.34390***

***Full citation:** “Sex disparities in the incidence of 21 cancer types: quantification of the contribution of risk factors.” Sarah S. Jackson, Morgan A. Marks, Hormuzd A. Katki, Michael B. Cook, Noorie Hyun, Neal D. Freedman, Lisa L. Kahle, Philip E. Castle, Barry I. Graubard, and Anil K. Chaturvedi. CANCER; Published Online: August 8, 2022 (DOI: 10.1002/cncr.34390).*

*Copyright © 2019 The Cochrane Collaboration. Published by John Wiley & Sons, Ltd., reproduced with permission.*

